# Outcomes and risks in palliative pancreatic surgery: an analysis of the German StuDoQ|Pancreas registry

**DOI:** 10.1186/s12893-022-01833-3

**Published:** 2022-11-11

**Authors:** Felix O. Hofmann, Rainer C. Miksch, Maximilian Weniger, Tobias Keck, Matthias Anthuber, Helmut Witzigmann, Natascha C. Nuessler, Christoph Reissfelder, Jörg Köninger, Michael Ghadimi, Detlef K. Bartsch, Werner Hartwig, Martin K. Angele, Jan G. D’Haese, Jens Werner

**Affiliations:** 1 Department of General, Visceral and Transplantation Surgery, Ludwig-Maximilians-University Hospital Munich, Marchioninistrasse 15, 81377 Munich, Germany; 2 Department of Surgery, University Clinic Schleswig-Holstein Campus Luebeck, Luebeck, Germany; 3 Department of General, Visceral and Transplantation Surgery, University Hospital Augsburg, Augsburg, Germany; 4 Department of General and Visceral Surgery, Dresden-Friedrichstadt General Hospital, Dresden, Germany; 5 Department of General and Visceral Surgery, Munich Clinic Neuperlach, Munich Municipal Hospital Group, Munich, Germany; 6 Department of Surgery, Universitätsmedizin Mannheim, Medical Faculty Mannheim, Heidelberg University, Mannheim, Germany; 7 DKFZ-Hector Cancer Institute at University Medical Center Mannheim, Mannheim, Germany; 8 Department of General Surgery, Klinikum Stuttgart, Stuttgart, Germany; 9 Department of General, Visceral and Pediatric Surgery, University Medical Center Goettingen, Goettingen, Germany; 10 Department of Visceral-, Thoracic- and Vascular Surgery, Philipps-University Marburg, Marburg, Germany; 11 Department of General, Visceral and Oncologic Surgery, Evangelisches Krankenhaus, Duesseldorf, Germany

**Keywords:** Pancreatic ductal adenocarcinoma, Explorative surgery, Palliative surgery, Biliary bypass, Gastroenteric bypass, Registry analysis

## Abstract

**Background:**

Non-resectability is common in patients with pancreatic ductal adenocarcinoma (PDAC) due to local invasion or distant metastases. Then, biliary or gastroenteric bypasses or both are often established despite associated morbidity and mortality. The current study explores outcomes after palliative bypass surgery in patients with non-resectable PDAC.

**Methods:**

From the prospectively maintained German StuDoQ|Pancreas registry, all patients with histopathologically confirmed PDAC who underwent non-resective pancreatic surgery between 2013 and 2018 were retrospectively identified, and the influence of the surgical procedure on morbidity and mortality was analyzed.

**Results:**

Of 389 included patients, 127 (32.6%) underwent explorative surgery only, and a biliary, gastroenteric or double bypass was established in 92 (23.7%), 65 (16.7%) and 105 (27.0%). After exploration only, patients had a significantly shorter stay in the intensive care unit (mean 0.5 days [SD 1.7] vs. 1.9 [3.6], 2.0 [2.8] or 2.1 [2.8]; P < 0.0001) and in the hospital (median 7 days [IQR 4–11] vs. 12 [10–18], 12 [8–19] or 12 [9–17]; P < 0.0001), and complications occurred less frequently (22/127 [17.3%] vs. 37/92 [40.2%], 29/65 [44.6%] or 48/105 [45.7%]; P < 0.0001). In multivariable logistic regression, biliary stents were associated with less major (Clavien–Dindo grade ≥ IIIa) complications (OR 0.49 [95% CI 0.25–0.96], P = 0.037), whereas—compared to exploration only—biliary, gastroenteric, and double bypass were associated with more major complications (OR 3.58 [1.48–8.64], P = 0.005; 3.50 [1.39–8.81], P = 0.008; 4.96 [2.15–11.43], P < 0.001).

**Conclusions:**

In patients with non-resectable PDAC, biliary, gastroenteric or double bypass surgery is associated with relevant morbidity and mortality. Although surgical palliation is indicated if interventional alternatives are inapplicable, or life expectancy is high, less invasive options should be considered.

**Supplementary Information:**

The online version contains supplementary material available at 10.1186/s12893-022-01833-3.

## Background

Pancreatic ductal adenocarcinoma (PDAC) is the fourteenth most common malignancy worldwide [1], but will be the second most frequent cause of cancer-related death by 2030 in industrialized countries [2, 3]. Resection is the precondition for cure and should be in combination with systemic treatment the goal in each patient [4, 5]. However, less than 20% of patients with PDAC present in primarily resectable stages [6].

In non-resectable locally advanced or metastatic PDAC, histopathology should be obtained by endoscopic or transcutaneous biopsy, and systemic treatment started as the therapy of choice [4, 5]. Biliary obstruction requires drainage that can be achieved interventionally by endoscopic retrograde cholangiopancreatography (ERCP) with stenting, or percutaneous transhepatic cholangiodrainage (PTCD) and subsequent internalization [7, 8]. Gastroduodenal obstruction should be treated endoscopically only in exceptional cases since stent dislocation and recurrent obstruction are common [4, 5, 9]. For the remaining patients, and after failure of less invasive methods, palliative bypass surgery may be necessary [4, 8, 10–13].

Metastases or locally advanced disease are discovered during laparotomy or laparoscopy in up to 40% of PDAC patients deemed resectable beforehand [14]. Then, palliative procedures need to be considered. Often, biliary or gastroenteric bypasses or both are established [4, 15, 16].

Only few studies have investigated morbidity and mortality among patients with PDAC after non-resective surgery [17, 18]. Reported morbidity rates range between 28 and 56%, with varying conclusions drawn by the authors [15, 19–21]: Some recommend a “watch-and-wait”-strategy instead of prophylactic bypass surgery [20], some conclude that bypass surgery should be avoided in high-risk populations [15], and others favor a selective approach considering the individual patient or the institution’s prerequisites [19, 21, 22]. Considering the morbidity after bypass surgery, and the improvements of endoscopic palliation, the balance of advantages and disadvantages of bypass procedures during laparoscopy or laparotomy is crucial.

Based on the German pancreatic surgery registry StuDoQ|Pancreas, the present study analyzes real-world morbidity and mortality after non-resective surgery in patients with PDAC and identifies associated risk factors.

## Methods

### StuDoQ|Pancreas registry

Data from the pancreatic surgery registry StuDoQ|Pancreas of the German Society for General and Visceral Surgery (DGAV) were retrospectively analyzed. StuDoQ|Pancreas is a prospectively maintained registry for pancreatic surgery established in September 2013 for the national assessment of quality and risk factors in pancreatic surgery in Germany [23]. At the time assessed by this study, more than 60 institutions were contributing to the registry, and 10 to 20% of all pancreatic surgeries in Germany were registered [24]. Pseudonymized data from the participating centers are prospectively entered using a web-based tool, undergoing automatic plausibility control. Validation by cross-checking with institutional medical data is part of the annual certification process. The informed consent and data safety concept were approved by the Society for Technology, Methods, and Infrastructure for Networked Medical Research (TMF) [23]. The present study was deemed for exemption by the institutional review board of the medical faculty of the LMU University of Munich (20–384 KB).

### Data extraction and patients

**Fig. 1:**
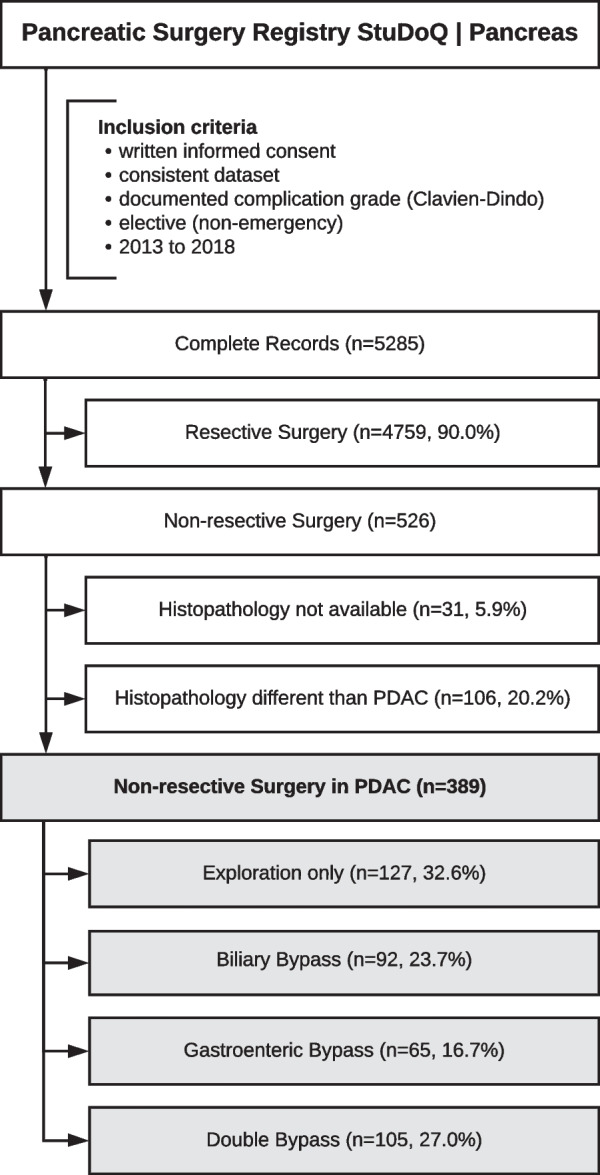
Study profile

All patients who provided written informed consent at the specific study site and underwent elective surgery between 2013 and 2018 were assessed for eligibility. Cases with inconsistencies in the dataset or undocumented complication status (Clavien–Dindo grade) were excluded (Fig. 1). All patients with histopathologically confirmed PDAC who underwent non-resective surgery (exploration, biliary bypass, gastroenteric bypass or double [biliary plus gastroenteric] bypass) either scheduled as palliative or when non-resectability turned out upon exploration were included.

### Data analysis

Beyond the surgical procedure, sex, age, BMI, biliary stent, ASA category, the localization of the tumor, the presence of liver metastases, and preoperative serum markers (bilirubin, CA19-9, and CEA) were obtained. Complications occurring after index surgery during the patient’s stay in the hospital were recorded, and dichotomized according to the Clavien–Dindo classification [25] into minor (grade ≤ II) and major (grade ≥ IIIa). Furthermore, the length of surgery, the length of stay in the intensive care unit (ICU) and in the hospital, the 30-day mortality, the 30-day readmission rate, the recommendation of palliative chemotherapy by the postsurgical multidisciplinary tumor board, and its actual start were recorded.

### Statistics

Variables were tested for normality by analyzing histograms and with Shapiro–Wilk tests. Normal distributions were reported in mean and standard deviations and compared using T-tests or ANOVA. Non-normal distributions were reported in median and inter-quartile ranges and compared using Wilcoxon rank-sum tests or Kruskal–Wallis tests. Categorical variables were compared using Fisher’s exact tests or Chi-squared tests. Missing values were not included into testing. When testing multiple times, P-values were adjusted with Hommel correction.

The association between potential risk factors and the occurrence of major complications (Clavien–Dindo grade ≥ IIIa) was explored in univariable logistic regression. Due to the limited number of events in our dataset, data reduction and variable selection were necessary for multivariable logistic regression. Thus, based on prior knowledge and independently from significance-levels in univariable analysis, we included into our full model fit the different types of bypass surgery (exploration vs. biliary/gastroenteric/double bypass), preoperative biliary stenting as a well-known risk factor in pancreatic surgery [26, 27], the presence of liver metastases characterizing more advanced and metastasized disease, and the ASA category to adjust for differences in the general health status of the investigated patients. The influence of having a biliary stent on the risk of complications of different variants of surgery was analyzed in logistic regression with interaction testing.

For regression analyses, missing data were imputed with multivariable imputations by chained equations assuming that they were missing at random using all relevant and available data (age, sex, BMI, biliary stent, ASA category, localization of the tumor, presence of liver metastases, preoperative bilirubin, preoperative CA19-9, preoperative CEA, surgery time, surgical procedure, major complication, postsurgical stay in ICU and in hospital). In 145/389 (37.3%) patients at least one variable was missing (Additional file 1: Fig. S1), therefore imputations were repeated 37 times. Results were checked numerically and visually for plausibility (Additional file 1: Figs. S2–S5). Logistic regression analyses were performed for all imputed datasets individually, and the results pooled.

The statistical analysis was performed using R version 4.2.1 (2022-06-23) [28] within RStudio version 2022.07.1 + 554 (Additional file 1: Table S1). Significance-level was set at 0.05 and all tests were conducted two-sided.

## Results

### Patients’ characteristics

The current study includes 389 patients with histopathologically confirmed PDAC who underwent palliative, non-resective procedures (Fig. 1). Of these, 127/389 (32.6%) underwent exploration only, 92/389 (23.7%) received a biliary bypass, 65/389 (16.7%) a gastroenteric bypass, and 105/389 (27.0%) the combination of both (double bypass).

The type of surgery depended highly on the localization of the tumor: If the tumor was located in the pancreatic head, biliary bypasses (alone or in combination with a gastroenteric bypass) were more frequently established (183/313, 58.5% vs. 14/76, 18.4%, P < 0.0001). Patients with tumors of the pancreatic body instead, underwent more frequently exploration only or gastroenteric bypass surgery (42/54, 77.8% vs. 150/335, 44.8%, P < 0.0001). In patients with PDAC of the pancreatic tail, exploration only was more common (15/18, 83.3% vs. 112/371, 30.2%, P < 0.0001).

Biliary bypass surgery (alone or in combination with a gastroenteric bypass) was performed more frequently in patients with higher preoperative bilirubin levels (median 3.9 mg/dl [IQR, 0.9 to 11.5] vs. 0.8 [0.4 to 1.9]; P < 0.0001), and in patients who underwent biliary stenting preoperatively (76/114, 66.7% vs. 121/275, 44.0%, P < 0.0001) (see also Biliary stenting). Other baseline parameters were well balanced between the different palliative surgical procedures (Table 1).

**Table 1:** Patients’ characteristics

	All non-resected	Exploration only	Biliary bypass	Gastroenteric bypass	Double bypass	P-value
	n = 389/389 (100%)	n = 127/389 (32.6%)	n = 92/389 (23.7%)	n = 65/389 (16.7%)	n = 105/389 (27.0%)	
Age, median [IQR]	68 [60 to 75]	66 [57 to 74]	69 [58 to 77]	67 [61 to 76]	69 [63 to 74]	0.452
Sex						0.088
Female	163 (41.9%)	48 (37.8%)	48 (52.2%)	22 (33.8%)	45 (42.9%)	
Male	226 (58.1%)	79 (62.2%)	44 (47.8%)	43 (66.2%)	60 (57.1%)	
BMI, median [IQR], kg/m^2^	24.2 [21.8 to 27.1]	24.0 [21.6 to 26.8]	25.2 [22.6 to 28.1]	23.6 [21.7 to 25.8]	24.1 [21.3 to 27.8]	0.059
Biliary stent	114 (29.3%)	21 (16.5%)	37 (40.2%)	17 (26.2%)	39 (37.1%)	**0.0002**
ASA						0.186
1	4 (1.0%)	–	–	1 (1.5%)	3 (2.9%)	
2	145 (37.3%)	47 (37.0%)	37 (40.2%)	20 (30.8%)	41 (39.0%)	
3	231 (59.4%)	78 (61.4%)	55 (59.8%)	41 (63.1%)	57 (54.3%)	
4	9 (2.3%)	2 (1.6%)	–	3 (4.6%)	4 (3.8%)	
Localization						** < 0.0001**
Head	313 (80.5%)	81 (63.8%)	87 (94.6%)	49 (75.4%)	96 (91.4%)	
Body	54 (13.9%)	31 (24.4%)	5 (5.4%)	11 (16.9%)	7 (6.7%)	
Tail	18 (4.6%)	15 (11.8%)	–	2 (3.1%)	1 (1.0%)	
Other	4 (1.0%)	–	–	3 (4.6%)	1 (1.0%)	
Liver metastases						
Present	140 (36.0%)	48 (37.8%)	31 (33.7%)	25 (38.5%)	36 (34.3%)	0.658
Absent	204 (52.4%)	62 (48.8%)	55 (59.8%)	31 (47.7%)	56 (53.3%)	
Unknown	45 (11.6%)	17 (13.4%)	6 (6.5%)	9 (13.8%)	13 (12.4%)	
Bilirubin, median [IQR], mg/dl	1.3 [0.6 to 9.0]	0.7 [0.4 to 2.7]	4.1 [0.8 to 12.3]	0.9 [0.5 to 1.6]	3.3 [0.9 to 10.8]	** < 0.0001**
Elevated (> 1.1 mg/dl)	202 (51.9%)	42 (33.1%)	63 (68.5%)	24 (36.9%)	73 (69.5%)	** < 0.0001**
Normal (≤ 1.1 mg/dl)	177 (45.5%)	81 (63.8%)	29 (31.5%)	38 (58.5%)	29 (27.6%)	
Unknown	10 (2.6%)	4 (3.1%)	–	3 (4.6%)	3 (2.9%)	
CA19-9, median [IQR], U/ml	384 [73 to 1421]	375 [66 to 1756]	426 [92 to 999]	327 [53 to 1340]	380 [96 to 1406]	0.967
Elevated (> 37 U/ml)	268 (68.9%)	89 (70.1%)	71 (77.2%)	42 (64.6%)	66 (62.9%)	0.898
Normal (≤ 37 U/ml)	59 (15.2%)	22 (17.3%)	13 (14.1%)	9 (13.8%)	15 (14.3%)	
Unknown	62 (15.9%)	16 (12.6%)	8 (8.7%)	14 (21.5%)	24 (22.9%)	
CEA, median [IQR], ng/ml	4.2 [2.3 to 10.5]	4.0 [2.0 to 10.6]	4.6 [2.9 to 10.6]	4.6 [2.5 to 12.5]	4.2 [2.0 to 7.9]	0.702
Elevated (> 5 ng/dl)	115 (29.6%)	46 (36.2%)	29 (31.5%)	18 (27.7%)	22 (21.0%)	0.723
Normal (≤ 5 ng/dl)	159 (40.9%)	57 (44.9%)	43 (46.7%)	21 (32.3%)	38 (36.2%)	
Unknown	115 (29.6%)	24 (18.9%)	20 (21.7%)	26 (40.0%)	45 (42.9%)	

Baseline characteristics according to procedure. Continuous data are shown as median [interquartile range], categorical data are shown as absolute (relative). P-values were derived from Fisher’s exact test, Chi-squared test, or Kruskal–Wallis test; unknown values were excluded when testing for differences. *ASA* ASA physical status classification system, *BMI* body mass index, *CA19-9* tumor marker carbohydrate antigen 19-9, *CEA* tumor marker carcinoembryonic antigen, *IQR*, interquartile range

### Outcomes

The time necessary to perform exploration only was significantly shorter than to establish a gastroenteric, biliary, or double bypass (P < 0.0001, P = 0.002, or P < 0.0001), and gastroenteric bypass surgery took significantly shorter than biliary or double bypass surgery (P < 0.0001, or P < 0.0001). Patients who underwent exploration only had a significantly reduced length of stay in the ICU and in the hospital (both P < 0.0001) (Table 2).

**Table 2:** Outcomes

	All non-resected	Exploration only	Biliary bypass	Gastroenteric bypass	Double bypass	P-value
	n = 389/389 (100%)	n = 127/389 (32.6%)	n = 92/389 (23.7%)	n = 65/389 (16.7%)	n = 105/389 (27.0%)	
Duration surgery, median [IQR], min	169 [102 to 218]	94 [62 to 143]	198 [168 to 244]	142 [86 to 186]	203 [177 to 250]	** < 0.0001**
Stay inhouse, median [IQR], days	11 [7 to 16]	7 [4 to 11]	12 [10 to 18]	12 [8 to 19]	12 [9 to 17]	** < 0.0001**
Stay ICU, mean [SD], days	1.5 [2.8]	0.5 [1.7]	1.9 [3.6]	2.0 [2.8]	2.1 [2.8]	** < 0.0001**
Unknown	9 (2.3%)	9 (7.1%)	–	–	–	
Complication Clavien–Dindo						
None	253 (65.0%)	105 (82.7%)	55 (59.8%)	36 (55.4%)	57 (54.3%)	**0.004**
I	26 (6.7%)	5 (3.9%)	7 (7.6%)	7 (10.8%)	7 (6.7%)	
II	46 (11.8%)	8 (6.3%)	13 (14.1%)	9 (13.8%)	16 (15.2%)	
IIIa	22 (5.7%)	5 (3.9%)	5 (5.4%)	5 (7.7%)	7 (6.7%)	
IIIb	20 (5.1%)	3 (2.4%)	5 (5.4%)	3 (4.6%)	9 (8.6%)	
IVa	6 (1.5%)	–	4 (4.3%)	–	2 (1.9%)	
IVb	–	–	–	–	–	
V	16 (4.1%)	1 (0.8%)	3 (3.3%)	5 (7.7%)	7 (6.7%)	
Complication						
None	253 (65.0%)	105 (82.7%)	55 (59.8%)	36 (55.4%)	57 (54.3%)	**0.0001**
Minor (Clavien–Dindo < IIIa)	72 (18.5%)	13 (10.2%)	20 (21.7%)	16 (24.6%)	23 (21.9%)	
Major (Clavien–Dindo ≥ IIIa)	64 (16.5%)	9 (7.1%)	17 (18.5%)	13 (20.0%)	25 (23.8%)	
30-day mortality rate	19 (4.9%)	3 (2.4%)	3 (3.3%)	7 (10.8%)	6 (5.7%)	0.075
30-day readmission rate						
Readmission	31 (8.0%)	8 (6.3%)	8 (8.7%)	7 (10.8%)	8 (7.6%)	0.886
No readmission	336 (86.4%)	97 (76.4%)	84 (91.3%)	58 (89.2%)	97 (92.4%)	
Unknown	22 (5.7%)	22 (17.3%)	–	–	–	
Recommendation of CTx						
Yes	328 (84.3%)	106 (83.5%)	79 (85.9%)	54 (83.1%)	89 (84.8%)	0.919
No	34 (8.7%)	10 (7.9%)	10 (10.9%)	5 (7.7%)	9 (8.6%)	
Unknown	27 (6.9%)	11 (8.7%)	3 (3.3%)	6 (9.2%)	7 (6.7%)	
Starting CTx after recommendation						
Yes	123/328 (37.5%)	63/106 (59.4%)	24/79 (30.4%)	14/54 (25.9%)	22/89 (24.7%)	0.731
No	50/328 (15.2%)	21/106 (19.8%)	12/79 (15.2%)	6/54 (11.1%)	11/89 (12.4%)	
Unknown	155/328 (47.3%)	22/106 (20.8%)	43/79 (54.4%)	34/54 (63.0%)	56/89 (62.9%)	

Outcomes according to procedure. Continuous data are shown as median [interquartile range], categorical data are shown as absolute (relative). P-values were derived from Fisher’s exact test, Chi-squared test, or Kruskal–Wallis test; unknown values were excluded when testing for differences. *CTx* chemotherapy, *ICU* intensive care unit, *IQR* interquartile range, *SD* standard deviation

**Fig. 2:**
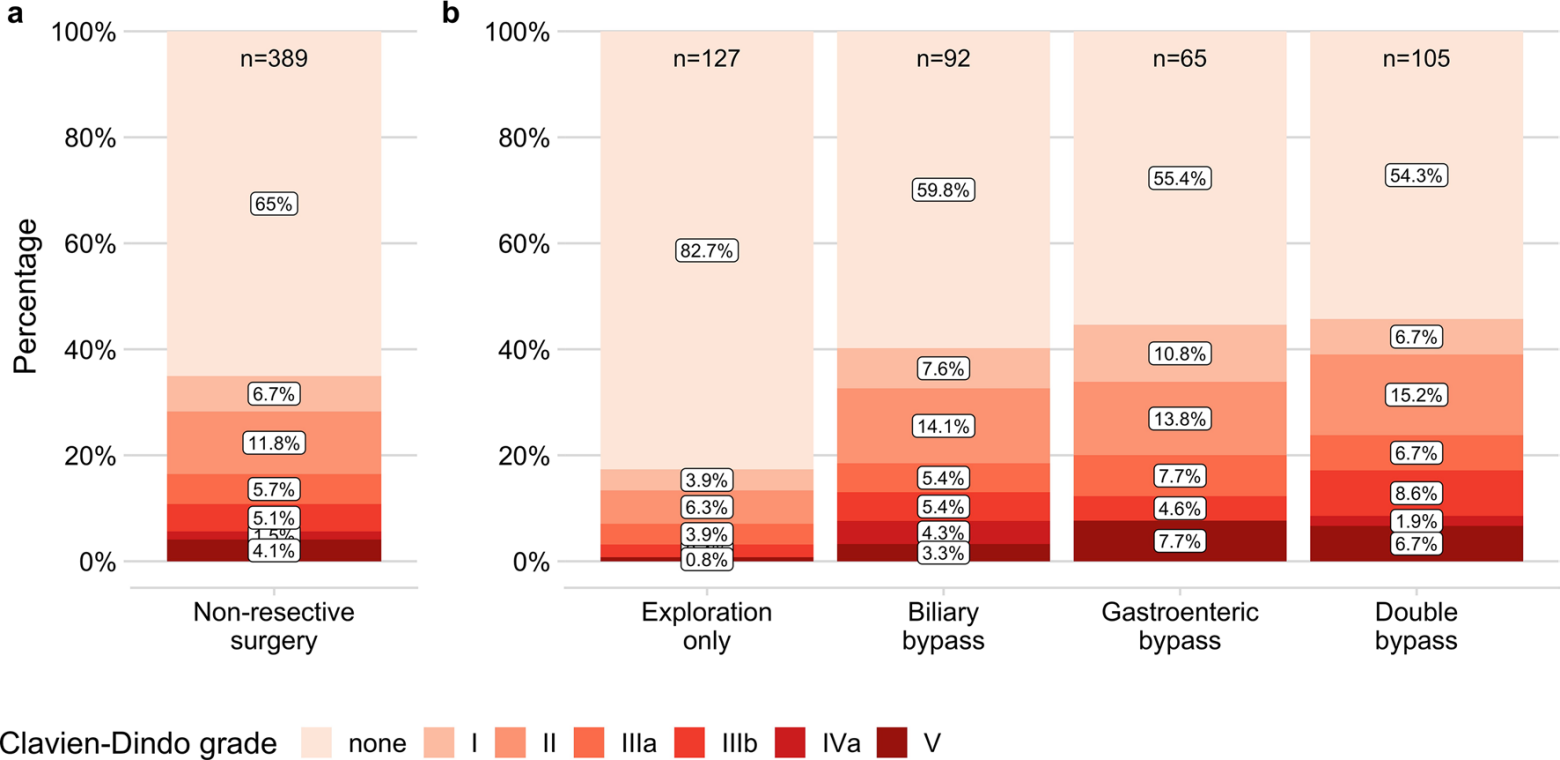
Rate of complications. Rate and grade of complications according to Clavien–Dindo in general (**a**) and regarding different types of non-resective surgery (**b**)

Complications occurred in 136/389 (35.0%), and major complications (≥ Clavien–Dindo grade IIIa) in 64/389 (16.5%) patients (Fig. 2a, Table 2). After exploration only, complications in general were less frequent than after biliary, gastroenteric or double bypass surgery (22/127, 17.3% vs. 37/92, 40.2% or 29/65, 44.6% or 48/105, 45.7%; P = 0.0008 or P = 0.0005 or P < 0.0001; Fig. 2b, Table 2). Also, we observed less major complications after exploration only than after biliary or double bypass surgery (9/127, 7.1% vs. 17/92, 18.5% or 25/105, 23.8%; P = 0.047 or P = 0.004, Fig. 2b, Table 2). The rate of major complications after exploration only and after gastroenteric bypass surgery did not differ significantly after adjusting for multiple testing (9/127, 7.1% vs. 13/65, 20.0%; P = 0.059). Also, the occurrence of deadly complications (Clavien–Dindo grade V) differed between the non-resective procedures in general (P = 0.032) but revealed no significant differences between individual groups after adjusting for multiple testing (Table 2).

In multivariable logistic regression adjusting for preoperatively placed biliary stents, ASA category, and liver metastases, establishing a biliary (OR 3.58 [95% CI 1.48 to 8.64], P = 0.005), gastroenteric (OR 3.50 [1.39 to 8.81], P = 0.008), or double bypass (OR 4.96 [2.15 to 11.43], P < 0.001) was associated with more major complications than exploration only.

### Postoperative chemotherapy

The postsurgical multidisciplinary tumor board recommended palliative chemotherapy in 328/389 (84.3%) patients, and in 123/328 (37.5%) it was actually administered. Patients started chemotherapy after sole exploration as frequently as after biliary, gastroenteric or double bypass surgery (P = 0.731) (Table 2). The rate of patients receiving palliative chemotherapy was significantly reduced in patients, who experienced any postoperative complication compared to those who did not (29/53, 54.7% vs. 94/120, 78.3%; P = 0.003).

### Biliary stenting

**Fig. 3:**
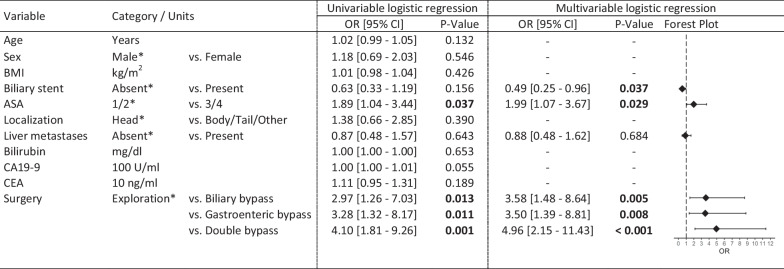
Risk of major complication. Predictors of major complications (Clavien–Dindo grade ≥ IIIa). Results were derived from univariable, and multivariable logistic regression based of the imputed dataset including all 389 patients. The reference category is marked by an asterisk (*). *ASA* ASA physical status classification system, *BMI* body mass index, *CA19-9* tumor marker carbohydrate antigen 19-9, *CEA* tumor marker carcinoembryonic antigen, *CI* confidence interval, *IQR* interquartile range, *OR* odds ratio

Patients with preoperatively placed biliary stents had higher bilirubin levels than patients without (median 2.2 mg/dl [IQR 0.9 to 5.4] vs. 1.0 [0.5 to 10.5]; P = 0.017), and biliary bypasses were more often established (76/114, 66.7% vs. 121/275, 44.0%; P < 0.0001). Preoperative biliary stenting was associated with a reduced risk of major complications in multivariable analysis (OR 0.49 [95% CI 0.25 to 0.96], P = 0.037; Fig. 3). The presence or absence of a biliary stent did not influence the risk of major complications associated with bypass surgery in interaction testing (P = 0.981).

### Subgroup of patients with normal bilirubin-levels

In the subgroup of 177 patients with normal bilirubin levels (≤ 1.1 mg/dl), complications in general were less frequent after exploration only than after biliary, gastroenteric or double bypass surgery (13/81, 16.0% vs. 12/29, 41.4% or 16/38, 42.1% or 14/29, 48.3%; P = 0.036 or P = 0.015 or P = 0.006). Also, we observed less major complications after exploration only than after biliary, gastroenteric, or double bypass surgery, however, the difference was only significant for the latter (7/81, 8.6% vs. 4/29, 13.8% or 6/38, 15.8% or 10/29, 34.5%; P = 0.952 or P = 0.714 or P = 0.013).

### Liver metastases

In the current dataset, bypass surgery (instead of exploration only) was performed regardless of whether liver metastases were present or not (92/140, 65.7% vs. 142/204, 69.6%; P = 0.481). Patients with liver metastases had major complications as frequently as patients without liver metastases (21/140, 15.0% vs. 34/204, 16.7%; P = 0.765), and the 30-day mortality rate was comparable (9/140, 6.4% vs. 6/204, 2.9%; P = 0.177).

## Discussion

The present study analyzed the real-world outcome after exploration and bypass procedures in non-resectable PDAC. The morbidity and mortality rates we found were remarkably high, consistent with previous findings [15–21, 29]. In particular, bypass surgery took longer than exploration alone, patients stayed longer in the intensive care unit and in the hospital, and complications were more frequent and more severe. This is in agreement with previous studies reporting lower morbidity after exploration only instead of bypass surgery (e.g., Bartlett et al. 12% vs. 20% or Williamsson et al. 31% vs. 67%) [15, 19–21]. Comparable to the trend in our study, an Italian registry study described mortality rates between 7.8% and 14.4% after bypass surgery, compared to 5.2% after explorative laparotomy and 2.6% after explorative laparoscopy [17].

The current study underlines that explorative surgery is associated with relevant complications, and the morbidity further increases when bypass surgery is performed. Our data did not yield baseline parameters identifying a specific high-risk subpopulation except the ASA category. Thus, the indication for a surgical bypass continues to be based on clinical judgement.

**Fig. 4:**
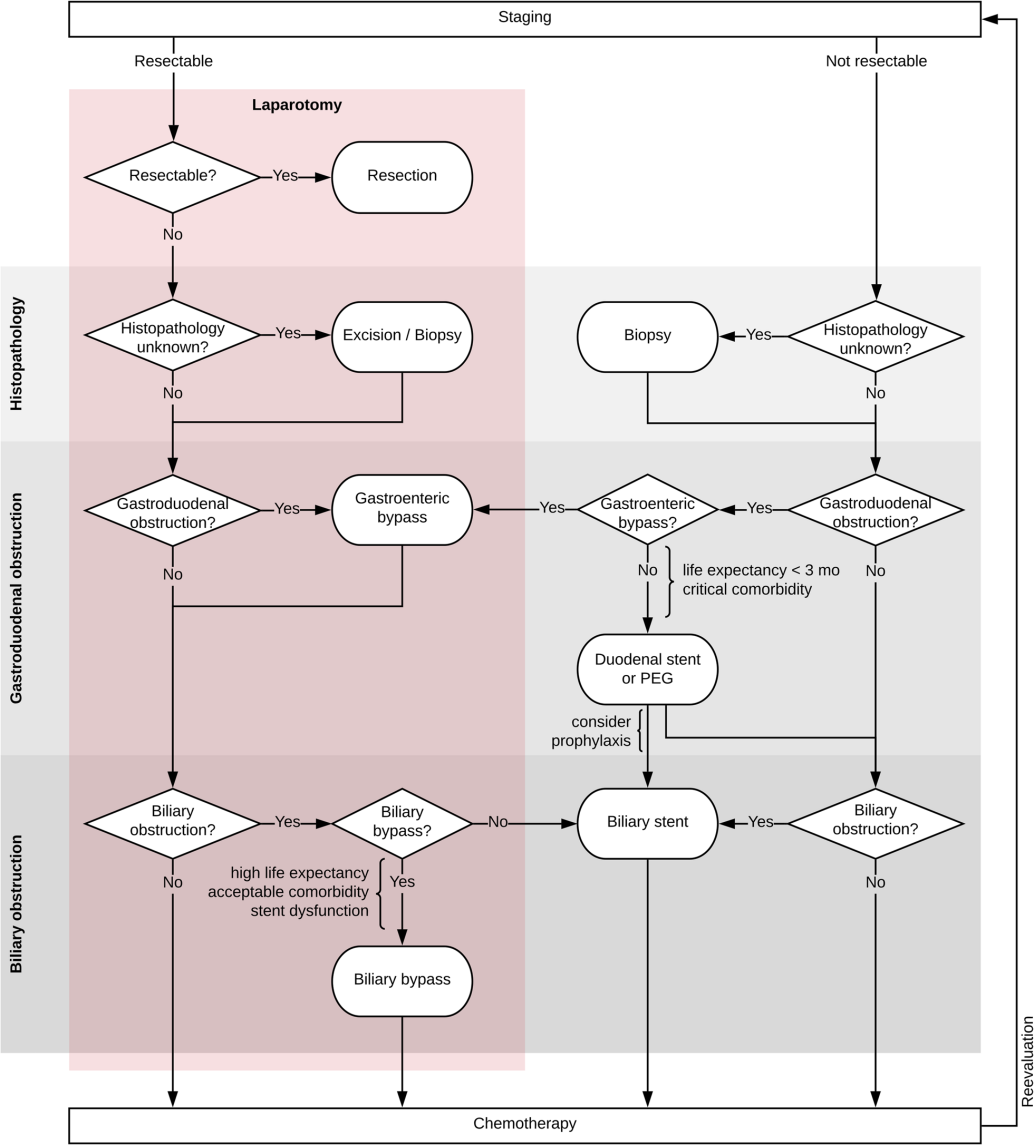
Recommended treatment algorithm. Recommended treatment algorithm of patients with pancreatic ductal adenocarcinoma. Rhombuses symbolize decisions, radiused rectangles symbolize interventions. Elements within the red box represent decisions or interventions during surgery. Grey boxes highlight different areas of concern such as obtaining histopathology, gastroduodenal obstruction, and biliary obstruction

Biliary obstruction occurs in 70–80% of patients with non-resectable pancreatic cancer someday [30]. Guidelines recommend palliation by endoscopic retrograde cholangiopancreaticograpy (ERCP) or percutaneous transhepatic cholangiodrainage (PTCD) with subsequent internalization instead of planned biliary bypass surgery due to reduced morbidity [4, 5, 8]. With advances such as the development of metal stents, disadvantages of endoscopic techniques such as the recurrence of biliary obstruction were reduced. After failure of ERCP, endoscopic ultrasound-guided biliary drainage (as choledochoduodenostomy or hepaticogastrostomy) can be discussed alternatively to PTCD. In non-resectable situations during explorative surgery however, the establishment of a biliary bypass may be considered [8]. High life expectancy (increasing the risk of stent obstruction and reintervention), low comorbidities, a high individual risk of biliary obstruction, or a history of stent dysfunction can indicate a biliary bypass [8, 12, 13], but should be balanced against the increased morbidity and mortality compared to exploration only (Fig. 4).

Of note, in our study the presence of a biliary stent was associated with reduced post-surgical morbidity, consistently with findings from Lyons et al. [21] and Spanheimer et al. [19]. The presence or absence of a biliary stent did not influence the risk of major complications associated with bypass surgery in interaction analysis. Still, in resective pancreatic surgery preoperative biliary stenting is associated with increased morbidity [26, 27]. Considering this, and in the light of the current study, the establishment of a prophylactic biliary bypass must be discussed carefully in patients with normal bilirubin-levels, and appears questionable in patients with functional, preoperatively placed biliary stent [31]. A recent study of Vreeland et al. supports this, showing that obstructive symptoms occurred frequently after exploration only, but could generally be treated without surgery [32].

Duodenal obstruction occurs in 6–25% of patients with non-resectable pancreatic cancer at some time [33, 34]. Endoscopic palliation by stenting is associated with shorter hospitalization times, early clinical improvement, and less morbidity than surgical gastrojejunostomy. However, stent dislocations and recurrent obstructions are common, and food tolerance is lower than after gastroenterostomy in the long term [9, 35]. Therefore, in patients with symptomatic duodenal obstruction, endoscopic palliation by stenting or percutaneous endoscopic gastrostomy (PEG) tube placement should be reserved to exceptions [4, 5, 9]. In patients with existing or impending duodenal obstruction, a life expectancy of 3 months or longer [9], and acceptable comorbidities, a gastroenteric bypass is indicated [4, 10, 11] and may also be established in non-resectable situations upon ongoing explorative surgery (Fig. 4). At tertiary centers with high endoscopic expertise and in selected patients, endoscopic ultrasound-guided gastroenterostomy can alternatively be discussed [36].

As morbidity and mortality of single (gastroenteric or biliary) and double (gastroenteric and biliary) bypass were comparable in our and prior studies [10, 11, 15, 34], one could argue, as soon as either a biliary bypass or a gastroenteric bypass is needed, a double bypass should be established. We still believe that the indication of each bypass should be reviewed individually as described earlier: First, endoscopic palliation of biliary obstruction is effective, and gastroduodenal obstruction requiring palliation less frequent. Second, although differences were not significant, in the gastroenteric and double bypass group deadly complications and 30-day mortality were most common, and in multivariable regression the risk of major complications highest. This might be consequence of both, a (often more advanced) tumor causing gastroduodenal obstruction, malnutrition and a reduced general health status, as well as the more invasive surgery in the double bypass group. Third, long-term outcomes from other studies support a “watch-and-wait” strategy: Espat et al. found that 151/155 (97.4%) patients with non-resectable PDAC at exploration did not require bypass surgery prior to death [37]. Lyons et al. report that 72/157 (46%) patients with non-resectable PDAC upon exploration required an additional invasive procedure in their further course of disease, independently whether a bypass was established or not [21]. Spanheimer et al. and Williamsson et al. report comparable rates of readmission or reinterventions [19, 20]. In summary, in these studies bypass surgery did not reduce the need of (endoscopic or surgical) reinterventions.

Patients with resectable and borderline resectable tumors should undergo surgery aiming for resection. Despite modern diagnostic staging, small metastases often remain undetected until explorative surgery [38]. Also, especially in the era of neoadjuvant chemotherapy, the predictive value of the image-based evaluation of local resectability is limited [39]. In patients with radiologically occult metastatic disease, diagnostic laparoscopy can reduce the surgical trauma. Sell et al. found, that these patients started palliative chemotherapy more quickly, and their overall survival was improved compared to patients undergoing exploratory laparotomy [40]. Still, the optimal definition of the subpopulation of patients benefiting from diagnostic laparoscopy remains controversial [41–43].

However, and in concordance with the current study, any reduction of the surgical trauma might improve the outcomes of patients with non-resectable PDAC. Thus, diagnostic laparoscopy in patients with high risk of metastatic disease, reservation of palliation to symptomatic patients, and preference of minimally invasive methods when appropriate, could in combination reduce procedural morbidity, increase the rate of patients receiving palliative chemotherapy, and accelerate its start. Future studies should explore the optimal combination, sequencing, and timing of diagnostic and therapeutic measures to maximize our patients’ cumulative quality of life [31, 44].

Admittedly, this study has limitations due to its design and the underlying registry. The registry does not specify whether non-resectability was known prior to surgery or turned out upon exploration. Patients undergoing explorative laparotomy in curative intention might be otherwise healthier than patients requiring planned bypass surgery. Pencovich et al. for instance describe a 30-day mortality of 16.6% in patients who underwent bypass surgery in purely palliative intention [22]. However, in the current study also in the subgroup of patients with preoperatively normal bilirubin-levels—suggesting that biliary bypass surgery was conducted prophylactically upon intraoperative detection of non-resectability—the morbidity was significantly increased after bypass surgery and comparable to the whole study population. Future studies should compare the outcomes of exploration only and bypass surgery in the subgroup of patients with tumors deemed resectable beforehand. Also, some potentially interesting data were often not available (e.g., survival beyond 30 days, postoperative quality of life, postoperative interventions, and reoperation due to biliary or duodenal obstruction) and could therefore not be analyzed. Still, the current study quantifies the real-world morbidity and mortality associated with bypass surgery in patients with non-resectable PDAC. This can help to properly balance the risks and benefits of palliative pancreatic surgery against those of less invasive alternatives.

## Conclusion

Having no reasonable option of resection during exploration will continue to play a role in pancreatic surgery. In this situation, the establishment of a biliary or gastroenteric bypass is associated with a relevant risk of complications. Thus, bypass surgery is indicated if less invasive alternatives are inapplicable or life expectancy is high, however, it should be up to careful clinical judgement in experienced pancreatic centers considering less invasive options.

## Supplementary Information

**Additional file 1: Table S1.** Software. **Fig S1.** Missingness and Missingness Patterns. **Fig S2. **Box-and-whisker plots of imputed values. **Fig S3.** Density plot of imputed values. **Fig S4.** Strip plot of imputed values. **Fig S5. **Mean and standard deviation of imputed values.

## Data Availability

The data that support the findings of this study are part of the StuDoQ|Pancreas registry and were provided by the German Society for General and Visceral Surgery (DGAV). Thus, they are not publicly available. Access can be requested from the German Society for General and Visceral Surgery (DGAV, http://www.dgav.de/studoq.html).

## Abbreviations

ASA: ASA physical status classification system
BMI: Body mass index
CA19-9: Tumor marker carbohydrate antigen 19-9
CEA: Tumor marker carcinoembryonic antigen
CI: Confidence interval
ERCP: Endoscopic retrograde cholangiopancreaticograpy
ICU: Intensive care unit
IQR: Interquartile range
OR: Odds ratio
PEG: Percutaneous endoscopic gastrostomy
PDAC: Pancreatic ductal adenocarcinoma
PTCD: Percutaneous transhepatic cholangiodrainage
