# Supplementary material for: Outcomes and risks in palliative pancreatic surgery: an analysis of the German StuDoQ|Pancreas registry
Source: BMC Surg. 2022 Nov 11;22:389. doi: 10.1186/s12893-022-01833-3 (PMC9652845; doi:10.1186/s12893-022-01833-3)

Outcomes and risks in palliative pancreatic surgery – an analysis of the German StuDoQ|Pancreas registry

ADDITIONAL DATA, STATISTICS

Table S1 Software

The statistical analyses were done using R version 4.2.1 (2022-06-23) – "Funny-Looking Kid" created and distributed by the R Core Team (https://www.R-project.org/) within RStudio 2022.07.1+554 – "Spotted Wakerobin". The following packages were used:

Package	Version	Usage
cowplot	1.1.1	Graphics
data.table	1.14.3	Data management
ggplot2	3.3.6	Graphics
grid	4.2.1	Graphics
lattice	0.20-45	Graphics
mice	3.14.0	Multiple Imputations by Chained Equations
VIM	6.1.1	Imputation analysis

Figure S1 Missingness and Missingness Patterns

Proportion of missing values (red) in the full dataset used for regression analysis on the left and missingness patterns of values in the full dataset and frequencies on the right. Based on the number of cases with at least one missing value 145/389 (37.3%), the number of permutations was defined.[1]

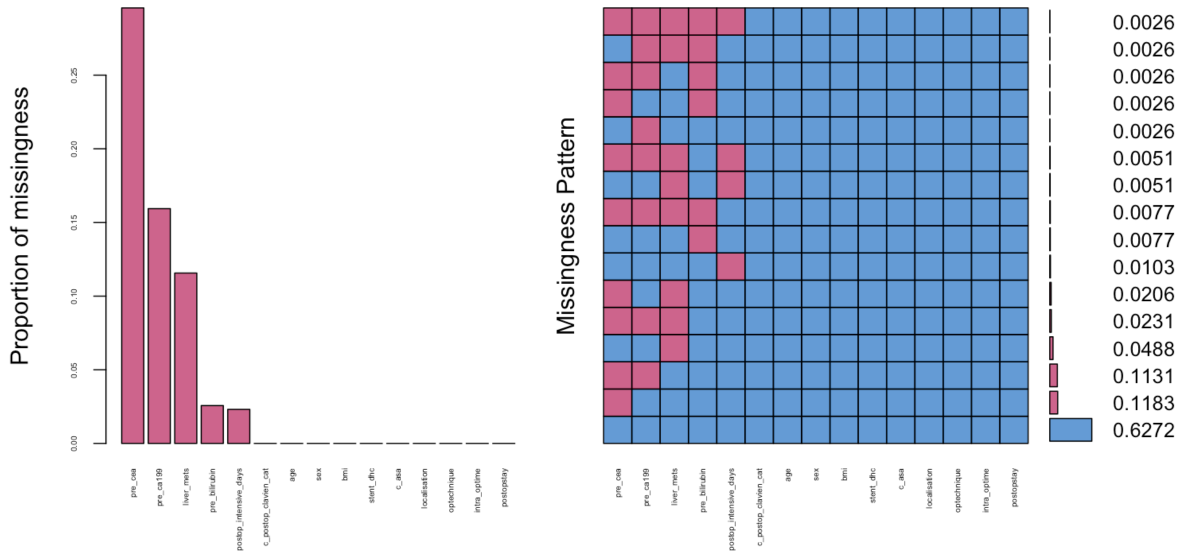

[1] Harrell FE: Regression Modeling Strategies, 2nd Edition, Springer Series in Statistics, Springer, Chan / Heidelberg / New York / Dordrecht / London, 2015.

Figure S2 Box-and-whisker plots of imputed values

Box-and-whisker plots of original, observed values (blue) and imputed variants (m = 37) of missing values by imputation and category (presurgical bilirubin, presurgical CA19-9, presurgical CEA and postoperative stay at the intensive care unit in days).

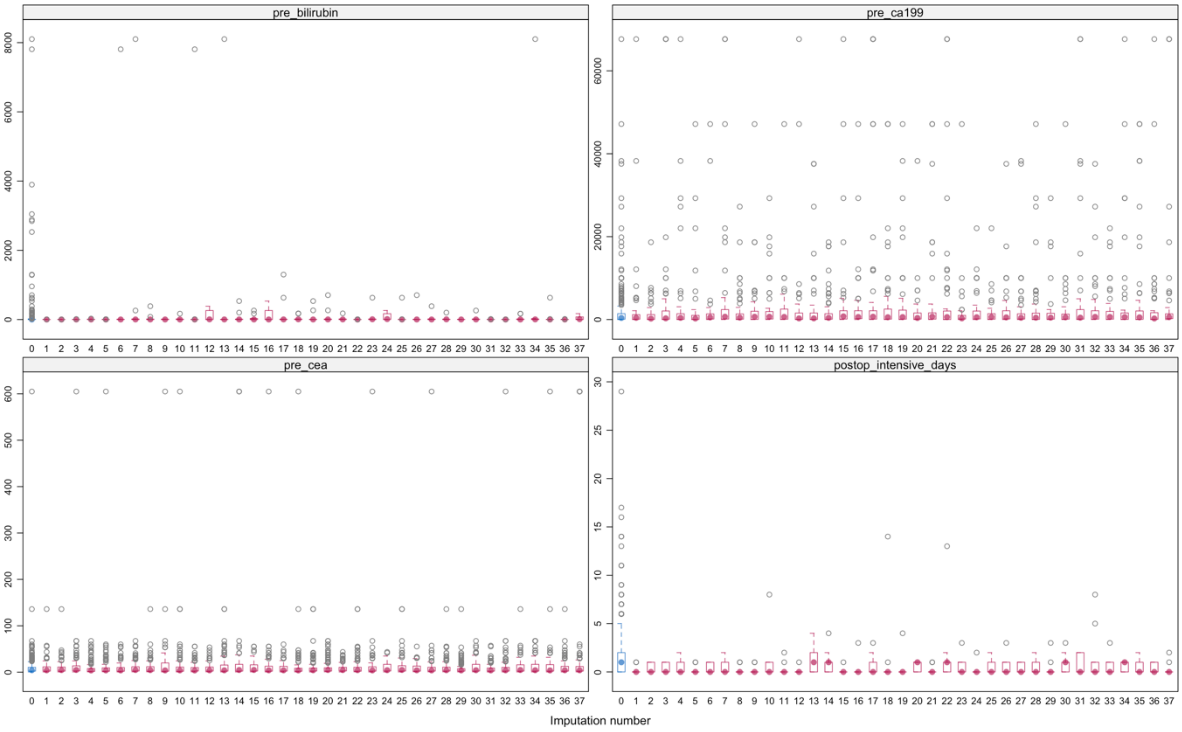

Figure S3 Density plot of imputed values

Density plot of original, observed values (blue) and imputed variants (m = 37) of missing values by value and category (presurgical bilirubin, presurgical CA19-9, presurgical CEA and postoperative stay at the intensive care unit in days).

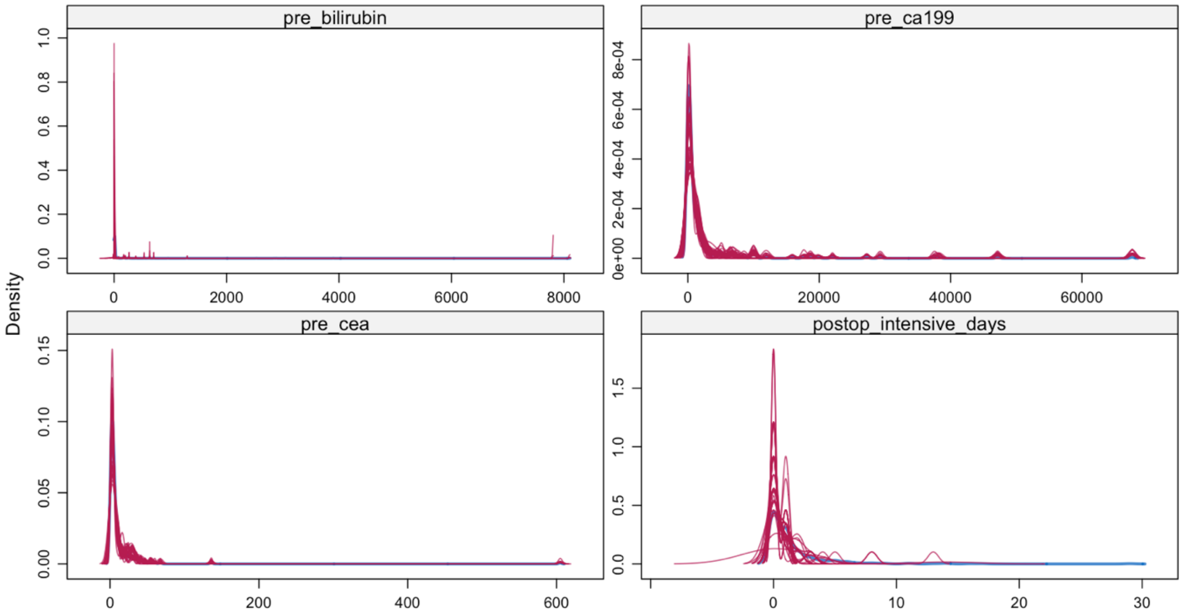

Figure S4 Strip plot of imputed values

Strip plot of original, observed values (blue) and imputed variants (m = 37) of missing values by imputation number and category (presurgical bilirubin, presurgical CA19-9, presurgical CEA and postoperative stay at the intensive care unit in days).

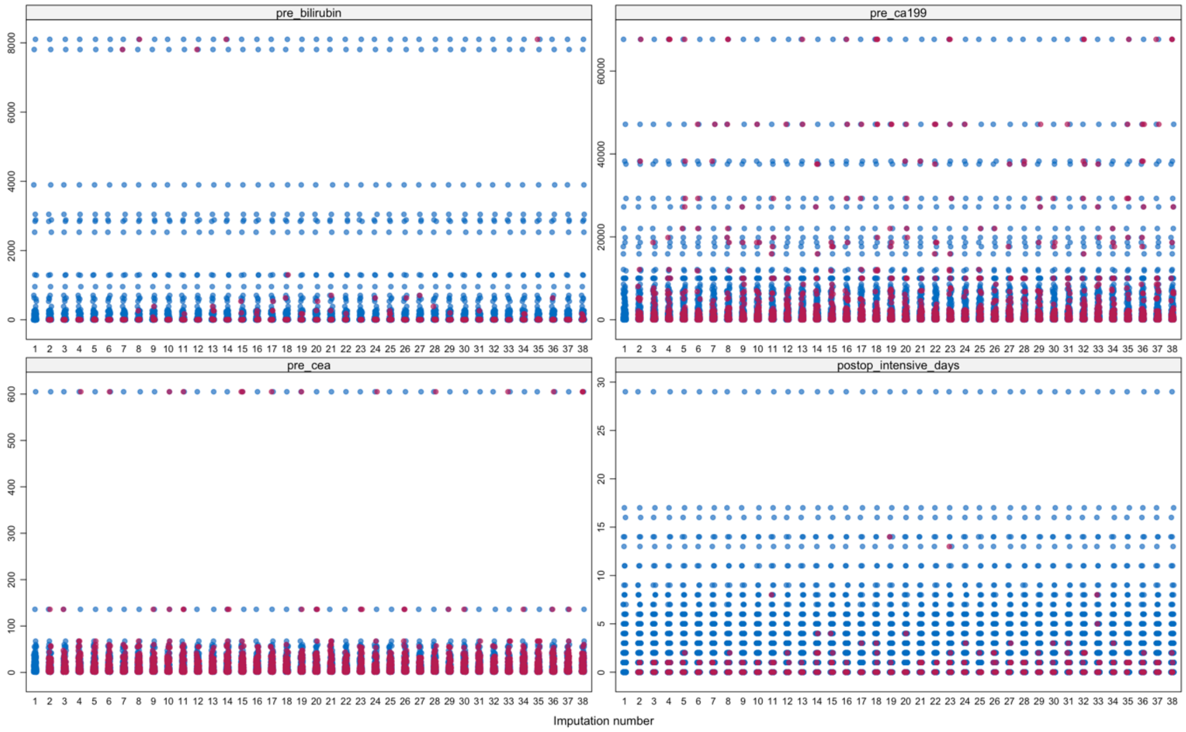

Figure S5 Mean and standard deviation of imputed values

Plots of the mean and standard deviation of the imputed values against the iteration number for each of the 5 replications. The lines intermingle, cross each other and do not show any trend. Therefore, systematic errors causing a trend between different imputations are unlikely.

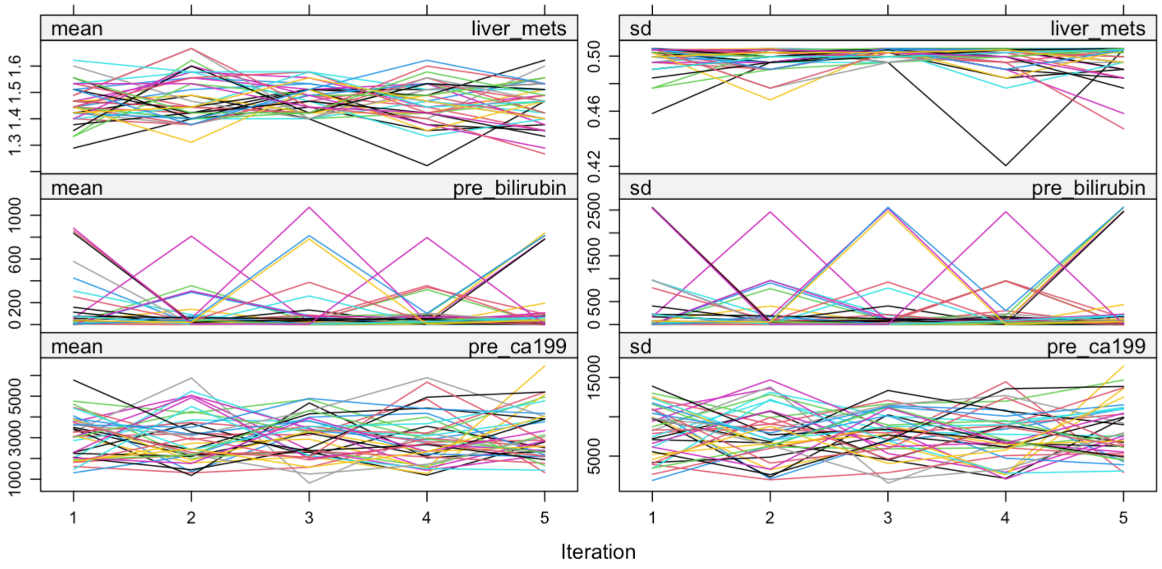

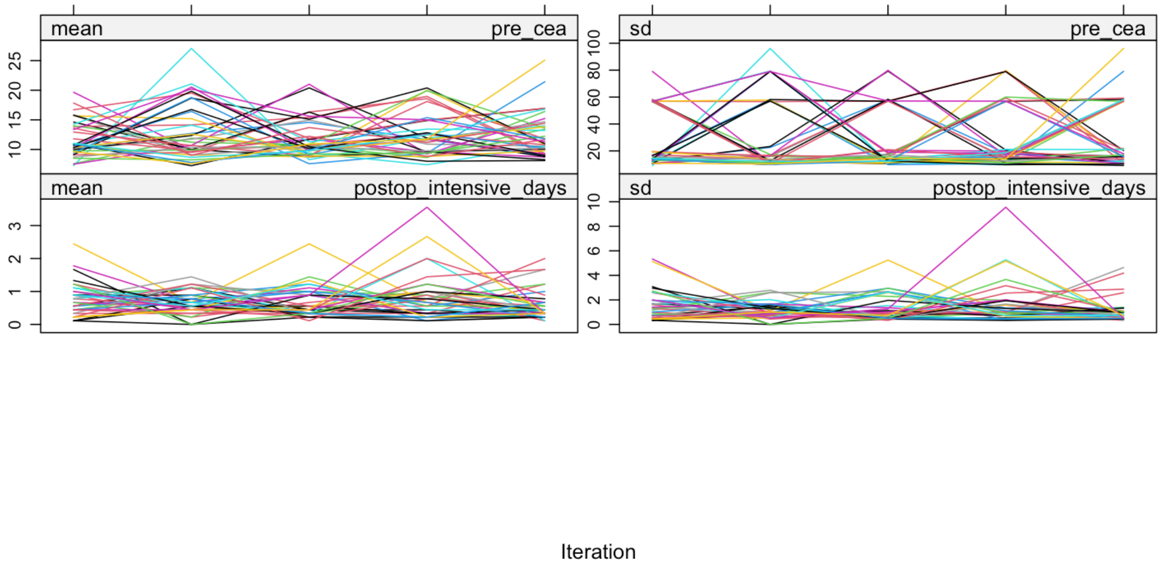

Supplement: Supplementary file 1 — Additional file 1: Table S1. Software. Fig S1. Missingness and Missingness Patterns. Fig S2. Box-and-whisker plots of imputed values. Fig S3. Density plot of imputed values. Fig S4. Strip plot of imputed values. Fig S5. Mean and standard deviation of imputed values. [file 12893_2022_1833_MOESM1_ESM.pdf]
